# Deep learning assisted multi-omics integration for survival and drug-response prediction in breast cancer

**DOI:** 10.1186/s12864-021-07524-2

**Published:** 2021-03-24

**Authors:** Vidhi Malik, Yogesh Kalakoti, Durai Sundar

**Affiliations:** DAILAB, Department of Biochemical Engineering and Biotechnology, Indian Institute of Technology (IIT) Delhi, New Delhi, India

**Keywords:** Multi-omics integration, Deep learning, Feature selection, Survival outcomes and drug response prediction

## Abstract

**Background:**

Survival and drug response are two highly emphasized clinical outcomes in cancer research that directs the prognosis of a cancer patient. Here, we have proposed a late multi omics integrative framework that robustly quantifies survival and drug response for breast cancer patients with a focus on the relative predictive ability of available omics datatypes. Neighborhood component analysis (NCA), a supervised feature selection algorithm selected relevant features from multi-omics datasets retrieved from The Cancer Genome Atlas (TCGA) and Genomics of Drug Sensitivity in Cancer (GDSC) databases. A Neural network framework, fed with NCA selected features, was used to develop survival and drug response prediction models for breast cancer patients. The drug response framework used regression and unsupervised clustering (K-means) to segregate samples into responders and non-responders based on their predicted IC50 values (Z-score).

**Results:**

The survival prediction framework was highly effective in categorizing patients into risk subtypes with an accuracy of 94%. Compared to single-omics and early integration approaches, our drug response prediction models performed significantly better and were able to predict IC50 values (Z-score) with a mean square error (MSE) of 1.154 and an overall regression value of 0.92, showing a linear relationship between predicted and actual IC50 values.

**Conclusion:**

The proposed omics integration strategy provides an effective way of extracting critical information from diverse omics data types enabling estimation of prognostic indicators. Such integrative models with high predictive power would have a significant impact and utility in precision oncology.

**Supplementary Information:**

The online version contains supplementary material available at 10.1186/s12864-021-07524-2.

## Background

Breast cancer has ranked among the most prevalent cancer type with a rate as high as 25.8 per 100,000 women in the Indian subcontinent [1]. Global and local studies have also reported a gradual increase in cancer-associated mortality in the region [2–4]. These metrics suggest an urgent need to devise robust knowledge-based prognostic systems that can generate phenotypic estimates for an individual. To address this issue, personalized medicine aims to provide the most effective treatment strategy based on the patient’s medical history, genomic characteristics, and response to therapy [5, 6]. Substantial genomic characterization has been conducted in the past decade to support the idea, leading to clinically relevant molecular subtyping [7–9]. Still, out of all the pharmaceutical agents pitched in clinical setups, only about 15% demonstrate sufficient safety and potency to gain any sort of regulatory consent [10, 11]. This implies the limitations in the current understanding of cancer complexity and the need for models that efficiently simulate the diversity of human tumor biology in a preclinical arrangement. With the advent of high-throughput data profiling technologies in the past decade, there is an opportunity for us to improve our understanding of the multi-layered molecular basis of cancer.

Large scale collaborative efforts such as The Cancer Genome Atlas (TCGA) and International Cancer Genome Consortium have led to numerous reports related to interim analyses of gene expression, somatic mutation, copy number variation (CNV) and protein expression data in the literature [12–16]. While it has allowed us access to a massive set of curated data, it is essential to address the long-standing bottleneck of omics integration to understand cancer prognosis and phenotype better. Multi-omics data integration has emerged as a promising approach for the prediction of clinical outcomes and identification of biomarkers in several cancer studies [17–20]. Modeling of survival and drug response clinical outcomes in cancer research can prove as steppingstones in the direction of personalized therapy. Omics integration allows us to analyze the human genome at multiple levels of complexity simultaneously and extract meaningful conclusions. Linear prediction models for such analysis often break down due to the steep dimensionality and heterogeneity associated with omics datasets. Hence, a refined integrative approach to handle these diverse datasets coherently is required.

Here, we address the challenge of building robust multi-omics integration based neural network models to predict clinical outcomes and response of an individual to a panel of 100 drugs. Neighbourhood component analysis (NCA) based feature selection algorithm was employed separately on each omics data to select high weighted features that were then fed into neural network-based classifier and regressor model to build multi-omics based integrative survival and drug response prediction models for breast cancer. These type of multi-omics integration based prediction models will not only help the physicians make rational chemotherapeutic decisions but also to understand the driving nodes in the cancer machinery.

## Results

We trained breast cancer datasets from TCGA and GDSC to generate robust survival and drug response prediction models. We used 10-fold cross-validation for the survival prediction model and 5-fold cross-validation for drug response prediction model to better tune the hyperparameters. Ultimately, two neural network models were chosen to generate drug responses and survival estimates for the patients in validation sets. The corresponding performance metrics were calculated based on the losses incurred in the respective models.

### Multi-omics integration improves survival prediction in BRCA patients

The NCA selected 246 six-omics feature set along with clinical features like age, gender, days to the last follow-up, pathologic stage, the number of affected lymph nodes, tumor stage, lymph node metastasis, metastatic stage and histological type were fed into neural network-based survival prediction model to classify the patients into two classes, i.e., high-risk class and low-risk class. The feed-forward neural network model was trained with two hidden layers of 7 nodes in each layer and an output layer of two neurons to classify patients into two survival classes. 10-fold cross-validation of neural-network along with optimization of regularization term and hidden layers architecture was performed using BayesOpt. The final layout of the neural network model consisted of two hidden layers (with seven nodes) and two output classes with a regularization term set to 0.9999. After multiple iterations of Bayesian optimization, ‘trainscg’ was selected as a training function that adopted a scaled conjugant gradient method to update weights and bias; cross-entropy was used as the performance evaluation function.

The survival prediction model was able to classify the patients into two survival classes – high-risk and low-risk, with a prediction accuracy of 94% (Fig. S1A). The prediction accuracies of training, validation and test dataset were 93.5, 93.7 and 98.1%, respectively. This clearly signified that the overfitting of the neural network model was successfully avoided here. AUROC (Area Under the Receiver Operating Characteristics) value of 0.98 was observed for both the classes, i.e., low-risk and high-risk, that showed the ability of prediction model to classify patients into two classes (Fig. S1B) efficiently. The performance of the model was also evaluated by calculating various other parameters like sensitivity, specificity, precision, false-positive rate, F1 Score, Matthews Correlation Coefficient and Kappa (Table 1). The value of all the parameters showed good ability of the prediction model to distinguish between two survival classes.

**Table 1:** Performance of neural network-based classifier for survival prediction of BRCA patients

	Sensitivity	Specificity	Precision	False positive rate	F1 Score	Matthews Correlation Coefficient	Kappa
**Parameters**	0.95	0.92	0.93	0.07	0.94	0.87	0.87

External validation of the multi-omics integration-based survival prediction model was performed by using single-omics and five-omics dataset of TCGA BRCA patients that were excluded for the training of model due to unavailability of all six-omics data (Table 2). The performance of the model with single-omics data or five-omics data as input for validation was not comparable to the performance of our model. It was observed that six-omics integrated data was able to predict both high-risk and low-risk individuals with good prediction accuracy. However, when single-omics or five-omics data was given as input for external validation, the model was not able to predict high-risk individuals correctly due to class imbalance in dataset available for breast cancer. It was observed that single-omics input classified all individuals as low-risk class, therefore correctly predicting low-risk patients with 100% prediction accuracy, but failed to predict for high-risk class. Similarly, for five-omics input feed, the model was able to predict high-risk individuals correctly with prediction accuracy ranging from 0 to 10% only and that of low-risk individuals with prediction accuracies ranging from 83 to 100%. This showed that adding more layers of omics information would aid in better prediction. Integrating different omics data types improved the performance of the predictive models over the traditional single-omics approach as the highest accuracy was achieved with the model including all the omics-types.

**Table 2:** External validation prediction accuracy of our multi-omics integration-based survival prediction model for BRCA patients

External Validation with Single-omics data and clinical features as input to model			External validation with five-omics data and clinical features as input to model		
Datatype	Samples	Prediction accuracy	Datatype	Samples	Prediction accuracy
RNA	561	85.7%	five-omics data excluding Protein	59	78.0%
Protein	397	85.1%	five-omics data excluding Mutation	41	73.2%
Mutation	493	85.8%	five-omics data excluding miRNA	103	90.3%
miRNA	241	82.6%	five-omics data excluding Methylation	111	77.5%
CNV	548	85.8%	five-omics data excluding CNV	3	66.7%
Methylation	268	86.6%	five-omics data excluding RNA	0	

### Multi-omics signature predicts drug response in BRCA cell lines

The drug response prediction model was trained on BRCA cell lines for 212 drugs initially; however, some drugs were filtered out later due to poor performance of models for these drugs. The final regression model was trained for 42 cell lines and 100 drug molecules. The robustness of the regression model using the features optimally selected using NCA was demonstrated using various performance metrics. The optimal neural network regressor had two hidden layered architecture with 11 nodes in both the layers. Levenberg-Marquardt backpropagation, which is the fastest backpropagation algorithm, was used as a training function to propagate the losses incurred back to the network and reconfigure the weights. In addition to this, Bayesian optimization of the regularization term was performed with the final value set to 0.3743 with 5-fold cross-validation to avoid overfitting the model. Mean squared error (MSE) was used as a performance evaluation function of the neural network regression model. The drug response prediction regression model predicted IC50 values for each drug with MSE of 1.154 and an overall regression value of 0.92, which showed the linear relationship between predicted and actual IC50 values. This was followed by unsupervised clustering (K-means) of drug responses to segregate the samples into responders and non-responders based on their IC50 values. The clustered IC50 values for the first twenty drugs showed that a common threshold value for all of the drugs could not be used as each drug has its unique distribution of responses (Fig. S2). The best validation performance reported in terms of MSE as 0.66 is remarkable, considering the small number of datasets. Moreover, calculation of IC50 thresholds was also consistent among the two methods (K-means and waterfall) as quantified by a strong correlation of 0.91 (Fig. S3-B). However, the classification metrics lagged while using thresholds calculated by waterfall analysis (Fig. S4).

**Fig. 1:**
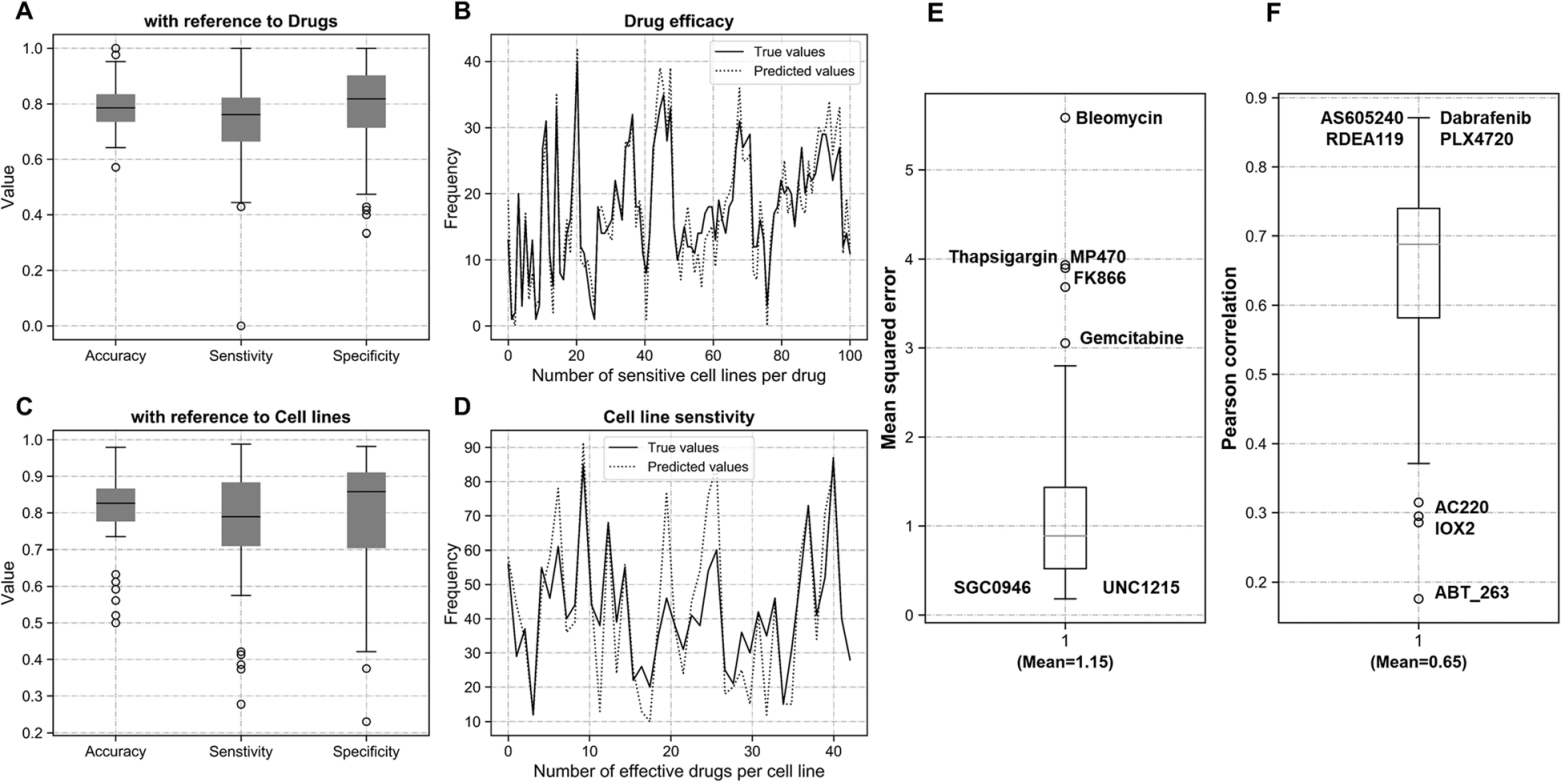
Performance of drug response model for BRCA cell lines. Box plot showing accuracy, sensitivity and specificity of model for all drugs (**a**) and all cell lines (**c**). Scatter plot showing frequency of (**b**) sensitive cell lines per drug molecule and (**d**) effective drugs for each cell line. **e** Mean squared error and (**f**) Pearson’s correlation for drug responses from the model for individual drugs

**Fig. 2:**
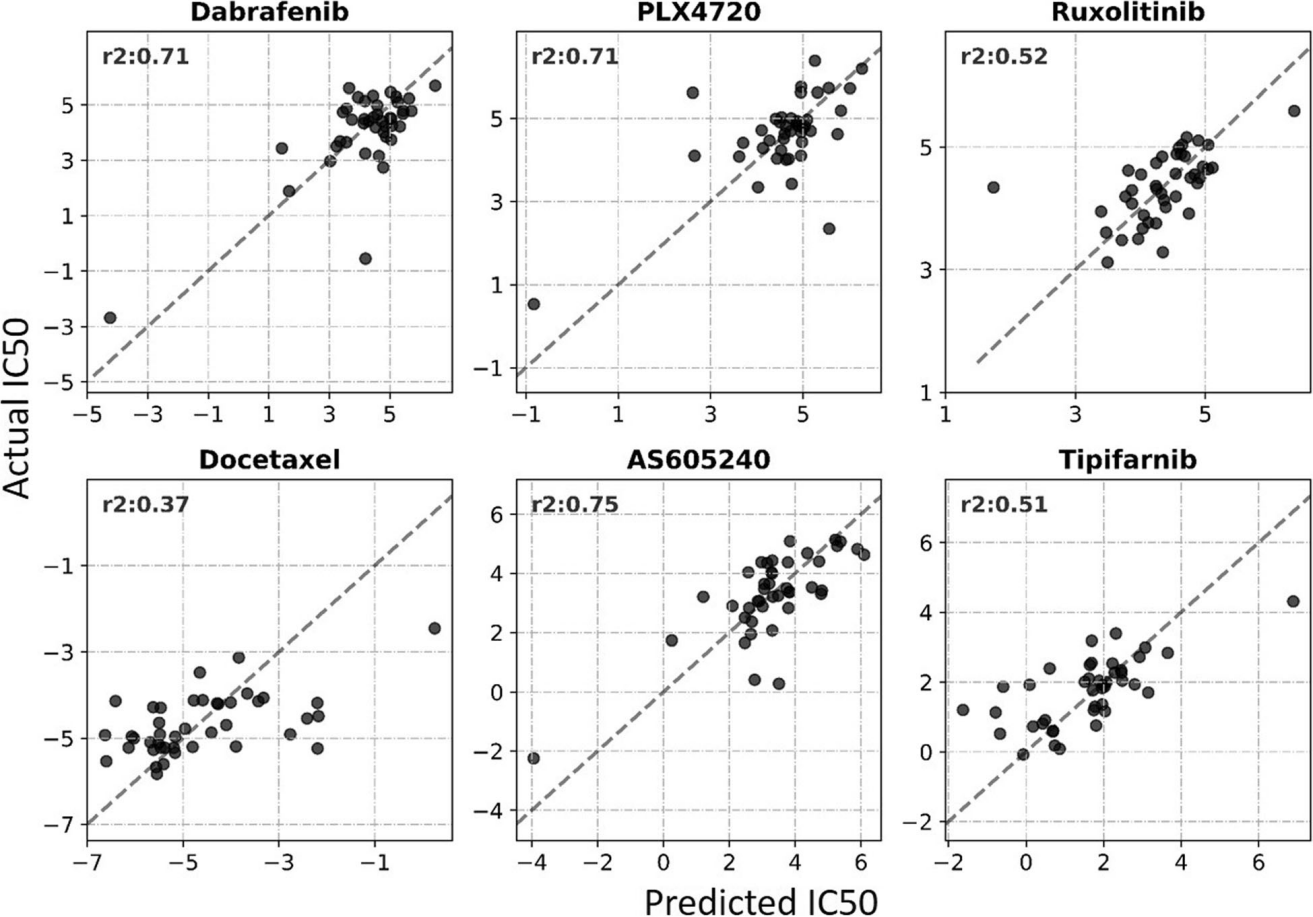
The drug response model, trained on the multi-omics profile of cell lines has the capacity to predict response for 100 drugs. The figure evaluates the performance for some of the prominent cancer drugs in terms of R squared performance measure (Note: For an ideal case, all the points would lie on a straight line y = x (dashed) with *r*2 = 1)

Drugs such as Dabrafenib, Mitomycin, Olaparib and Ruxolitinib performed exceptionally well on almost all the cell lines tested. Figure 1 shows the performance of drug response in terms of accuracy, specificity, and sensitivity corresponding to all the drugs as well as all the cell lines. It is evident from the results that most of the drugs performed at par or even outperform similar drug response prediction models [21]. These traditional methods employed Elastic Net and SVM models for drug response on GDSC datasets instead of Deep learning frameworks. Hence, their average sensitivity and specificity values were averaged around 0.75 and 0.78 respectively. Even with a large ensemble of tested drugs (100), the average sensitivity and specificity values reported here averaged around 0.80 (Fig. 1a and c). Individual drugs were analyzed for their contribution to the overall performance metrics that led to the discovery of certain outliers like Bleomycin, Gemcitabine, Thapsigargin, MP470 and FK866 (Fig. 1e-f). While these drugs negatively affected the model performance, drugs such as Dabrafenib, AS605240, RDEA119 and PLX4720 depicted exceptional correlation with the actual drug-responses across the test set (Fig. 1f and 2).

### Proposed model performs better than similar approaches

The proposed breast cancer survival and drug response prediction models were compared with one survival prediction method and two drug response prediction methods (Table 3). For survival prediction, a similar study on BRCA patients reported accuracy and AUC values of 0.73 and 0.79 respectively [22]. As a direct comparison, our proposed model performed significantly better for the same metrics with prediction accuracy of 0.94 and AUC value of 0.98.

**Table 3:** Comparison of the proposed survival and drug response prediction model with similar methods

**Survival Prediction**	**Accuracy**	**AUC**
C. Wang et al. [22]	0.79	0.93
Proposed method	**0.94**	**0.98**
**Drug response prediction**	**Docetaxel (AUC)**	**Gemcitabine (AUC)**
MOLI	0.67	0.71
SVM	0.63	0.69
Proposed method	**0.83**	**0.78**

On the other hand, SVM-based and late-integration based models have been extensively used to predict drug responses in cancer patients [23]. On similar lines, an SVM model was built *in-house* using NCA selected features for comparative analysis. SVM parameters were optimized using grid search on a range of cost and gamma that were adapted from a similar SVM based study [23]. A value of 10 for cost and 0.5 for gamma was found to be optimal for predicting drug responses. Similarly, MOLI was employed to predict drug responses for our datasets (https://github.com/hosseinshn/MOLI) [19]. However, only a subset of the drugs (Docetaxel and Gemcitabine) could be compared as MOLI was limited to only a few drugs. The proposed method was able to outperform the competition on both the instances, reinforcing the effectiveness of the proposed method (Table 3).

Moreover, to gauge the effectiveness of the proposed drug response model, a measure of external validation was necessary. Drug response data for TCGA breast cancer (BRCA) patients was available from a similar study [24]. TCGA identifiers and drug responses for four drugs (Vinblastine, Gemcitabine, Tamoxifen, Docetaxel) were extracted from the dataset. mRNAseq, methylation, CNV and miRNAseq data for the selected TCGA identifiers was processed and passed through the saved neural network. The predicted drug responses, binarized using previously calculated drug thresholds, were fairly accurate with about 0.79 accuracy for Docetaxel (24 patients) and 0.5 for Tamoxifen (11 patients). For Vinblastine and Gemcitabine, the dataset of single patient for each drug was available to compare predictions of developed drug response prediction model. The developed model was able to predict drug response for Vinblastine and Gemcitabine correctly. Therefore, considering that the initial model was trained on cell lines, the overall external validation accuracy of 0.73 is consistent with internal validation and reinforces the effectiveness of the proposed method.

### Biological significance of identified signature

Feature selection using NCA provided us with a set of genes that were weighted highly for their predictive potency. Therefore, Gene Set Enrichment Analysis (GSEA) was employed to calculate gene enrichment scores corresponding to every entity. Reactome knowledge database was used to carry out the analysis [25, 26]. Gene set screened from mRNA dataset for the survival prediction module revealed pathways and reactions that are critical for the patient’s survival (Table S2). TP53 dependent transcription regulation, gene expression and DNA damage response were among the most significantly enriched pathways among all data types. The identified signature of survival and drug response prediction was also combined and mapped onto KEGG pathways using DAVID functional annotation tool [27, 28]. The identified pathway mainly consisted of cancer pathways and all major pathways whose dysregulation is well reported in cancer (Table S3).

## Discussion

Robust classification of cancer patients into risk groups and having prior information about the possible drug responses will identify novel screening methods, prognostic factors, methods and perhaps guide the next steps in personalized therapies. In this study, the high prognostic accuracy of neural networks has been demonstrated owing to their capacity to model complex relationships among variables [29, 30].

For identification of probable prognostic biomarkers among the screened gene-pool, a ranking criterion was devised among the genes. The screening methodology (NCA) enabled us to rank the associated genes based on their predictive ability. Four genes, EFHD1, CDH1, PIK3CA and TP53, were identified by our feature selection algorithm that aid in prediction of both survival and drug response prediction of breast cancer patients. The role of these genes, to serve as prognostic/predictive biomarkers has already established in many cancer types (Table 4). EF-hand Domain Family Member D1 (EFHD1) is shown to be overexpressed in breast cancer and is reported to serve as a potent breast cancer-specific RNA signature [36]. Similarly, genetic and epigenetic alterations in E-Cadherin (CDH1) relates to aberrant expression and microsatellite instabilities in breast cancer patients have also been related to the incidence of breast cancer [37, 38]. Besides, Phosphatidylinositol 3-kinase (PIK3CA) and Tumor protein 53 (TP53) genes, which are two of the most mutated genes in breast cancer, were also shortlisted by the workflow [39, 40].

**Table 4:** Biological significance of gene set that aid in prediction of BRCA survival and drug response

GENE	NAME	Reported Biomarker function
EFHD1	EF-Hand Domain Family Member D1	Part of digital RNA resistance signature to predict response to breast cancer therapy [31]
CDH1	Cadherin 1	CDH1 structural alterations as novel prognostic biomarker in gastric cancer patients [32] CDH1 gene as a prognostic biomarker in hepatocellular carcinoma [33]
PIK3CA	Phosphatidylinositol-4,5-bisphosphate 3-kinase, catalytic subunit alpha	PIK3CA is a predictive biomarker for use of alpelisib and fulvestrant in BRCA patients [34]
TP53		mutant p53 as a possible therapeutic target and biomarker for breast cancer [35]

The drug response model captured the relationship between the patient’s multi-omics profile and well-known breast cancer drugs such as Dabrafenib (*r*2 = 0.71), Gemcitabine (*r*2 = 0.59) and (AS605240) PI3K inhibitor (*r*2 = 0.75) among others with a high degree of confidence (Fig. 2). In addition to the omics types included in the study, the approach can be theoretically scaled for the integration of other omics types such as proteomics. Ambiguous data remains to be a hurdle in the way of these models being clinically acceptable. For example, patients who die of an unrelated cause or have a sparse follow-up will have to be incorporated accordingly into the model. A few alternatives to mitigate this issue is reported in the literature, but none of them have yet been successful [41, 42].

## Conclusions

Survival statistics are one of the most important prognostic factors in breast cancer. However, it can be debated whether a response to therapy is also as detrimental to the patient’s ultimate treatment routine. Probing the potential of cumulative analysis of survival prediction and response to therapy could open doors for practical solutions in improving therapy in cancer. Global genomic profiling of cancer cell line panels and patient-derived samples have contributed a lot in building risk-classification models and suggesting novel therapeutic measures. However, a large pool of drug compounds has not been assessed over the potential of available genomics data. With an increase in biological resources that capture disease characteristics such as genotype, phenotype and their associations, novel strategies are required to efficiently process this information and reveal critical insights for the disease. Here, we employed late integrative deep learning frameworks for building survival and drug response prediction models that performed at par with existing individual solutions.

We conclude that an artificial deep neural network, which is trained on the multi-omics signature of an individual, in tandem with its clinico-pathological factors, can not only segregate individual into low-risk and high-risk subgroups but also assist in screening a pool of drugs based on the sensitivity values corresponding to the patient under observation. The results reinforce the idea that an integrative approach can make more accurate and personalized decisions for drug administration and general treatment strategy.

## Methods

### General workflow

**Fig. 3:**
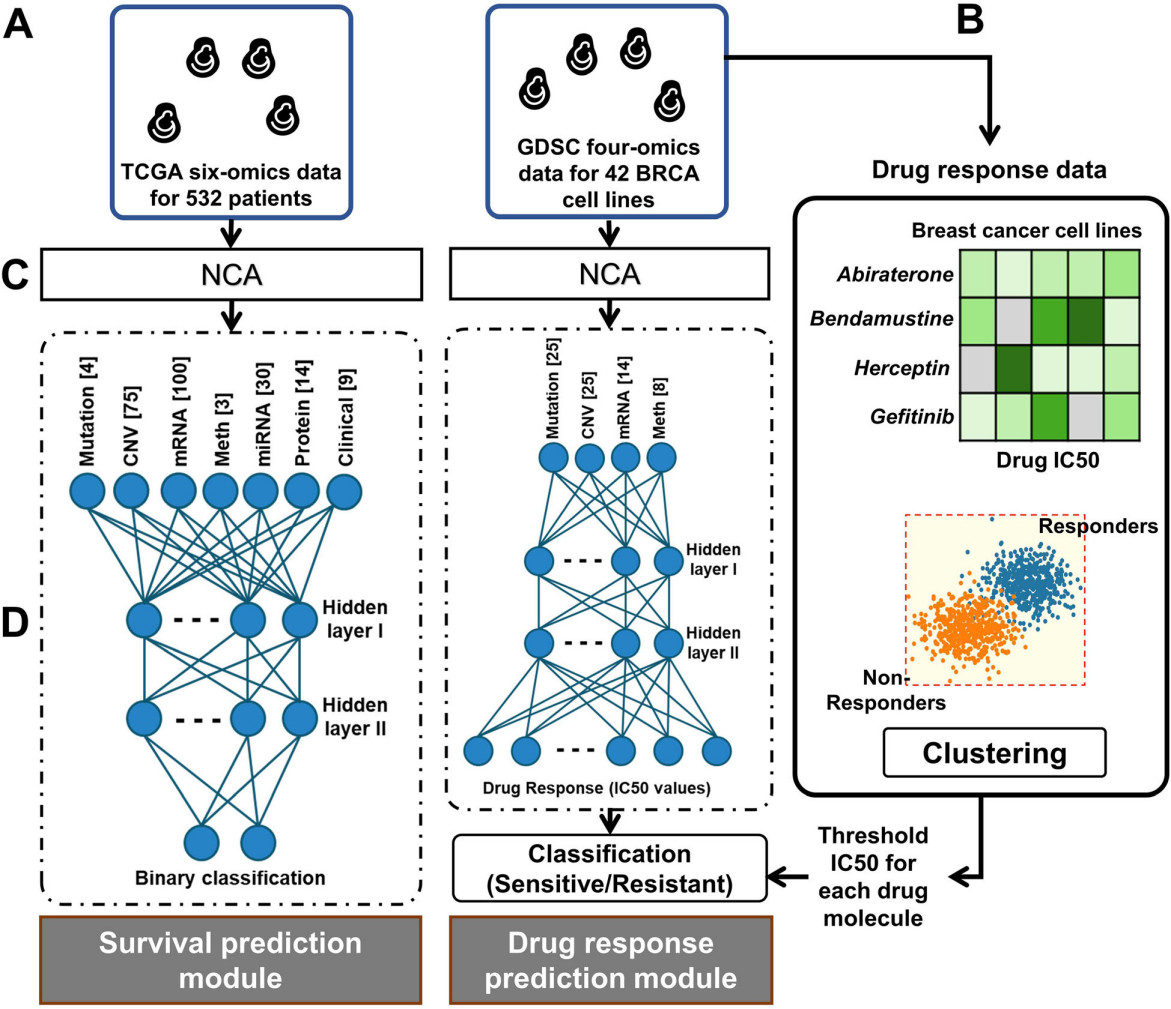
Schematic of the general pipeline followed for survival and drug response prediction task. The flowchart depicting various steps followed during prediction models development including (**a**) data retrieval, (**b**) drug response processing, (**c**) omics data processing and (**d**) training/optimizing deep neural networks

This workflow was designed to predict the survival outcome and drug response for a given BRCA patient, characterized by its multi-omics signature. The underlying assumption is data being independent and identically distributed. The workflow followed multiple feed-forward networks and dimensionality-reduction measures corresponding to every omics type. The features learned were clubbed together that served as an input to a regression and classification network for drug-response and survival prediction, respectively (Fig. 3).

### Datasets

Two major resources were used for the analysis. Datasets for breast invasive carcinoma (BRCA) patients were retrieved from TCGA, whereas GDSC was used to source multi-omics as well as drug-response datasets for BRCA cell lines [43]. GDSC was preferred among other sources due to its broad spectrum of screened drugs.

### Preprocessing TCGA breast cancer patient’s data

TCGA BRCA multi-omics datasets, along with their clinical information was available for more than 1000 patients, including 1089, 977, 1097, 1078, 1093 and 887 patient’s GISTIC2 CNV, mutation, methylation, miRNA, RNA and protein expression data respectively. The pre-processed TCGA dataset was obtained using FireBrowse utility (http://firebrowse.org). For RNA, z-scaled RSEM values of RNA expression were used and for miRNA log2-RPM values were retrieved. Protein expression and methylation data (β values) obtained from database were already scaled. Binary data was obtained for mutation of genes and GISTIC2 calculated CNV data was obtained directly from FireBrowse. The dataset was screened by filtering patients and features with more than 20% missing values. Further missing values in the omics dataset were imputed using R package impute [44]. An overlapping set of 314 patients was obtained for which all six-omics datasets along with their clinical information was available. The final processed data was observed to be class imbalanced. Therefore, an oversampling technique called Synthetic Minority Oversampling TEchnique (SMOTE) [45] was employed to balance the data that increases our sample set from 314 to 532.

### Preprocessing breast cancer cell line data obtained from GDSC

The breast cancer cell lines omics data and drug response data were retrieved from the GDSC database. Already preprocessed data for all cell lines was obtained from the GDSC database followed by filtration step to filter out other cancer cell lines data and only breast cancer cell lines data was retained for the analysis. Binary data was obtained for gene mutations and already pre-processed β-values were obtained for methylation of CpG islands. RMA normalized basal expression level was obtained for RNA data and copy number values were obtained ranging from − 1 to 1, where 0 indicates normal copy number, − 1 and + 1 indicates loss and gain of copy of genes respectively. The overlapping set of 43 cell lines were selected for those drug responses for which at least 80% of drugs and all four omics datasets, namely CNV, methylation, mutation and mRNA, were available. Similar preprocessing was done to remove cell lines and features having more than 20% missing values. This reduced our sample set to 42 cell lines only. Remaining missing values in omics and drug response data were imputed using the impute R package. The next filter for genes was applied to screen out genes for which omics data was not available in the TCGA BRCA dataset.

### Constructing representative gene sets

The high dimensionality of omics datasets remains a significant bottleneck in generating robust prediction models that are clinically relevant [46]. The goal of feature engineering here is to find an effective low-dimensional manifold of a given high dimensional dataset. Fortunately, biological processes are highly correlated and can be represented in a lower-dimensional sub-space [47, 48]. Many approaches, like Principal component analysis, Correspondence Analysis, Partial Triadic Analysis and Multiple co-inertia analysis, which are based on variance, correlation, inertia, eigenvalue among others have utilized this fact. They have been quite successful in this effort [49–51]. However, none of these commonly used approaches considers the effect of labels corresponding to the datasets. Due to this particular reason, we opted for Neighborhood Component Analysis (NCA), which is a supervised dimensionally reduction method for learning Mahal Nobis distance measure for k-nearest Neighbors [52].

Given an omics data set, *X* = *x*_1_, *x*_2_, *x*_3_, …, *x*_*n*_ ∈ *R*^*P*^ and corresponding class labels *c*_1_, *c*_2_, *c*_3_, …, *c*_*n*_, which is generally an *n* × *p* matrix with *n* observations (patients) and *p* variables (genes) corresponding to the measurements of mRNA and other omics datasets. NCA reduces the dimensions by restricting the quadratic distant metric to be low rank. The underlying distance metric can be defined as follows.

1 $$ d\left(x,y\right)={\left(x-y\right)}^T\left(z-y\right)={\left( Ax- Ay\right)}^T\left( Ax- Ay\right) $$

Also, Leave One Out performance is utilized as test data is not available during training under the following objective function.

2 $$ f(A)={\sum}_i{\sum}_{j\in {C}_i}{p}_{ij}={\sum}_i{p}_i $$

The ultimate features extracted corresponding to each omics type for building survival and drug response models are summarized in Table S1.

### Multi-omics feature integration

A single multi-omics representation of different omics datasets was generated by employing a late integration approach, where the learned features for each of the omics types were concatenated before being fed into the neural networks. For instance, three single-omics input with three *m* × *n* feature matrices will result in a single *m* × 3*n* representation matrix after integration.

### Survival prediction

A two-layer neural network model was constructed for the binary classification (survival) task using MATLAB ver. R2019b. Cross-entropy loss was employed for the optimization of the objective function. Losses were propagated back using scaled conjugate gradient backpropagation and the hyperparameters were optimized using Bayesian hyperparameter optimization (BayesOpt) [53]. Hyperbolic tangent, a symmetric activation function that provides mean-zero initial weights, was used in the hidden layers, followed by sigmoid activation at the output layer [41]. The output of the network that ranged from zero to one was used to infer the risk group as a categorical variable.

3 $$ CE=\left\{\begin{array}{c}-\log \left(f\left({s}_1\right)\right)\  if\ {t}_1=1\\ {}-\log \Big(1-\left(f\left({s}_1\right)\right)\  if\ {t}_1=0\end{array}\right. $$

where *t*_1_ = 1 denotes the assignment of *C*_1_ = *C*_*i*_ for the sample. The entire network and its parameters were optimized using grid search and Bayes-opt optimizer.

### Drug response prediction

Similar to the architecture of the survival prediction model, the drug response prediction neural network also had two hidden layers, followed by an output regression layer. The drug response model was trained on data points from 42 cell lines to predict drug responses of 212 drugs initially using MATLAB ver. R2020a (data not shown). However, with a limited model capacity due to small dataset, modelling large number of drug responses had an inverse effect on the performance, reflected in below-par metrics for many drugs. Therefore, another model was built for limited number of drugs that is reported in this study. This was done by eliminating drugs depicting an accuracy of less than 0.5. A total of 100 drugs fulfilled the criteria and the network architecture was modified to predict their drug responses.

The neural network was modelled as a regression problem to predict IC50 values. However, to binarize predicted IC50 to responses as sensitive or resistant, the original IC50 values were clustered into two classes using K-means clustering. It tries to make the inter-cluster points as similar as possible while trying to keep the clusters as far as possible under the objective function defined in eq. (4).

4 $$ J=\sum \limits_{i=1}^m\sum \limits_{k=1}^K{w}_{ik}{\left|\left|{x}^i-{\mu}_k\right|\right|}^2 $$

The threshold IC50 value between the two classes was saved for each drug and later used to compare and test the effectiveness of the drug response prediction model. In addition to K-means as a method to calculate thresholds, waterfall analysis was also performed. Our implementation of the waterfall analysis was similar to a previous approach [54, 55]. For each of the 100 drugs, IC50 values were sorted to generate a waterfall distribution (Fig. S5). If the distribution is non-linear (Pearson correlation coefficient to linear fit ≤0.95), the inflection point was calculated by first smoothening the curve with a gaussian filter, followed by analysing the differential. In case of a linear distribution (Pearson correlation coefficient to linear fit > 0.95), median IC50 was used instead. 43 drugs had a non-linear waterfall distribution (Fig. S3-A). Inflection points and medians were used as thresholds to segregate among sensitive and resistant cell lines.

### Hyperparameter optimization

BayesOpt and grid search was employed for tuning the parameters of the classification and regression neural network models. BayesOpt builds a probability model of the objective function to screen the best parameters to evaluate the model objective function [56]. For both drug response regression and survival classification tasks, hyperparameters corresponding to the objective functions were optimized using BayesOpt. The basic formulation is represented in eq. (5).

5 $$ {x}^{\ast }=\arg {\mathit{\min}}_{x\in \varkappa }P\left( score\ \right|\ x\Big) $$

where *P*(*y*| *x*) is the surrogate objective function (Mean Square Error or cross-entropy) and *x*^∗^ is the set of hyperparameters with the best model performance. It works by finding the parameters that correspond to the best performing surrogate function and using them on the actual objective function iteratively.

## Supplementary Information

**Additional file 1: Table S1.** Description of the total number of features for each dataset that were used for multi-omics data integration type for building survival and drug response models. **Table S2** REACTOME pathways mapped onto the screened genes that outlines the critical reactions and modules that modulates a patient’s survival. **Table S3** KEGG pathways mapped by identified signature of aberration for survival and drug response prediction model.

**Additional file 2: Figure S1.** Performance of multi-omics integration based neural network survival prediction model of BRCA patients. (A) Confusion matrix and (B) ROC plot of neural network prediction model.

**Additional file 3: Figure S2.** Violin plot showing the distribution of IC50 values of sensitive (blue) and resistant (orange) cell lines for the first 21 drugs.

**Additional file 4: Figure S3.** (A) Histogram depicting drugs with linear and non-linear correlation to a linear fit. (B) Correlation of IC50 thresholds calculated from the two methods (K-means and waterfall) shows that the two methods have consistent results.

**Additional file 5: Figure S4.** Performance of drug response model using thresholds from waterfall analysis. Box plot showing accuracy, sensitivity, and specificity of model for all drugs (A) and all cell lines (C). Scatter plot showing frequency of (B) sensitive cell lines per drug molecule and (D) effective drugs for each cell line.

**Additional file 6: Figure S5.** Waterfall distribution for 100 drugs under considerations. Blue lines depict IC50 thresholds as calculated by inflection point and median (threshold IC50 value is also depicted on each plot).

## Data Availability

All the codes and data used for the study can be accessed at https://github.com/TeamSundar/BRCA_multiomics.

## Abbreviations

AUROC: Area Under the Receiver Operating Characteristics
BRCA: Breast invasive carcinoma
CNV: Copy number variation
GDSC: Genomics of Drug Sensitivity in Cancer
GSEA: Gene Set Enrichment Analysis
MSE: Mean square error
NCA: Neighborhood component analysis
SMOTE: Synthetic Minority Oversampling TEchnique
TCGA: The Cancer Genome Atlas
