# Supplementary material for: Deep learning assisted multi-omics integration for survival and drug-response prediction in breast cancer
Source: BMC Genomics. 2021 Mar 24;22:214. doi: 10.1186/s12864-021-07524-2 (PMC7992339; doi:10.1186/s12864-021-07524-2)
Supplement: Supplementary file 1 — Additional file 1: Table S1. Description of the total number of features for each dataset that were used for multi-omics data integration type for building survival and drug response models. Table S2 REACTOME pathways mapped onto the screened genes that outlines the critical reactions and modules that modulates a patient’s survival. Table S3 KEGG pathways mapped by identified signature of aberration for survival and drug response prediction model. [file 12864_2021_7524_MOESM1_ESM.docx]

Deep learning assisted multi-omics integration for survival and drug-response prediction in Breast cancer

Vidhi Malik, Yogesh Kalakoti, and Durai Sundar^$^

DAILAB, Department of Biochemical Engineering and Biotechnology, Indian Institute of Technology (IIT) Delhi, New Delhi, India

^$^Corresponding author

**Table S1** **Description of the total number of features for each dataset that were used for multi-omics data integration type for building survival and drug response models.**

| **Omics Type** | **NCA Output**  **(Survival prediction model)** | **NCA Output**  **(Drug response prediction model)** |
| --- | --- | --- |
| CNV | 75 x 532 | 25 x 42 |
| mRNA | 100 x 532 | 14 x 42 |
| Methylation | 23 x 532 | 8 x 42 |
| Mutation | 4 x 532 | 25 x 42 |
| miRNA | 30 x 532 | - |
| Protein | 14 x 532 | - |

**Table S2 REACTOME pathways mapped onto the screened genes that outlines the critical reactions and modules that modulates a patient’s survival.**

| Pathway name | p-value | Reactions found | Submitted entities found |
| --- | --- | --- | --- |
| TP53 Regulates Transcription of DNA Repair Genes | 2.94209E-14 | 15 | POLR2B, ATM, BRCA1, TP53, ATR |
| Transcriptional Regulation by TP53 | 9.30478E-13 | 178 | POLR2B, PTEN, ATM, CHD4, BRCA1, TP53, ATR |
| Gene expression (Transcription) | 6.78575E-09 | 252 | POLR2B, CDH1, PTEN, ATM, CHD4, BRCA1, NUP98, ARID1A, TP53, BAP1, ATR, KDM6A |
| Generic Transcription Pathway | 2.51441E-07 | 196 | POLR2B, CDH1, PTEN, ATM, CHD4, BRCA1, ARID1A, TP53, BAP1, ATR |
| SUMOylation of DNA damage response and repair proteins | 5.72137E-07 | 8 | STAG2, BRCA1, NUP98, BAP1 |
| RNA Polymerase II Transcription | 9.9882E-07 | 242 | POLR2B, CDH1, PTEN, ATM, CHD4, BRCA1, ARID1A, TP53, BAP1, ATR |
| PIP3 activates AKT signalling | 2.14283E-06 | 45 | PIK3CA, PTEN, CHD4, BRCA1, PIK3R1, TP53, BAP1 |
| DNA Double-Strand Break Repair | 2.68484E-06 | 72 | ATM, BRCA1, BRCA2, TP53, BAP1, KDM6A, ATR |
| PTEN Regulation | 3.13342E-06 | 42 | PTEN, CHD4, BRCA1, TP53, BAP1 |
| Regulation of TP53 Activity | 4.06899E-06 | 69 | ATM, CHD4, BRCA1, TP53, ATR |
| Recruitment and ATM-mediated phosphorylation of repair and signaling proteins at DNA double strand breaks | 4.77956E-06 | 33 | ATM, BRCA1, TP53, BAP1, KDM6A |

Table S3 KEGG pathways mapped by identified signature of aberration for survival and drug response prediction model.

| KEGG Pathway | Gene Count | P value | Genes |
| --- | --- | --- | --- |
| Pathways in cancer | 20 | 3.90E-05 | DCC, FGF19, EGFR, DVL3, CTBP2, FGF7, BRAF, FLT3, TP53, BRCA2, CDH1, NFKB1, PTEN, FZD6, AKT1, CCND1, PIK3CA, PIK3R1, MAPK1, MAPK3 |
| PI3K-Akt signaling pathway | 17 | 1.00E-03 | EGFR, FGF19, AKT1, CCND1, FGF7, IL2RA, MCL1, TP53, PIK3CA, NFKB1, PDGFD, THBS1, PTEN, BRCA1, PIK3R1, MAPK1, MAPK3 |
| Proteoglycans in cancer | 15 | 6.90E-05 | AKT1, EGFR, CCND1, BRAF, LUM, TP53, ESR1, PIK3CA, THBS1, TIMP3, PIK3R1, PLAU, FZD6, MAPK1, MAPK3 |
| HTLV-I infection | 14 | 4.30E-05 | AKT1, DVL3, CCND1, IL2RA, MAP3K1, MAP2K4, TP53, PIK3CA, NFKB1, ATR, PIK3R1, ATM,, DLG1, FZD6 |
| Melanoma | 14 | 1.10E-08 | FGF19, AKT1, EGFR, CCND1, FGF7, BRAF, TP53, PIK3CA, CDH1, PDGFD, PTEN, PIK3R1, MAPK1, MAPK3 |
| MAPK signaling pathway | 14 | 2.10E-03 | FGF19, AKT1, EGFR, FGF7, BRAF, PAK2, MAP3K1, NF1, MAP2K4, TP53, NFKB1, HSPA1A, MAPK1, MAPK3 |
| Focal adhesion | 13 | 1.50E-03 | AKT1, EGFR, CCND1, BRAF, PAK2, MYLK3, PIK3CA, PDGFD, THBS1, PTEN, PIK3R1, MAPK1, MAPK3 |
| Ras signaling pathway | 13 | 3.00E-03 | FGF19, AKT1, EGFR, FGF7, PAK2, NF1, PIK3CA, NFKB1, PDGFD, PLA2G3, PIK3R1, MAPK1, MAPK3 |
| Prostate cancer | 12 | 9.10E-06 | AKT1, EGFR, CCND1, BRAF, TP53, PIK3CA, NFKB1, PDGFD, PTEN, PIK3R1, MAPK1, MAPK3 |
| Rap1 signaling pathway | 12 | 5.90E-03 | FGF19, AKT1, EGFR, FGF7, BRAF, PIK3CA, CDH1, PDGFD, THBS1, PIK3R1, MAPK1, MAPK3 |
| Regulation of actin cytoskeleton | 11 | 5.90E-03 | FGF19, EGFR, FGF7, BRAF, PAK2, MYLK3, PIK3CA, PDGFD, PIK3R1, MAPK1, MAPK3 |
| Pancreatic cancer | 11 | 7.30E-06 | AKT1, EGFR, CCND1, BRAF, TP53, BRCA2, PIK3CA, NFKB1, PIK3R1, MAPK1, MAPK3 |
| Hepatitis B | 11 | 2.00E-03 | AKT1, CCND1, MAP3K1, MAP2K4, TP53, PIK3CA, NFKB1, PTEN, PIK3R1, MAPK1, MAPK3 |
| Endometrial cancer | 11 | 9.40E-08 | AKT1, EGFR, CCND1, BRAF, TP53, PIK3CA, CDH1, PTEN, PIK3R1, MAPK1, MAPK3 |
| Glioma | 10 | 6.80E-05 | AKT1, EGFR, CCND1, BRAF, TP53, PIK3CA, PTEN, PIK3R1, MAPK1, MAPK3 |
| CML | 10 | 1.30E-04 | AKT1, CCND1, CTBP2, BRAF, TP53, PIK3CA, NFKB1, PIK3R1, MAPK1, MAPK3 |
| FoxO signaling pathway | 10 | 5.20E-03 | AKT1, EGFR, CCND1, BRAF, PIK3CA, PTEN, PIK3R1, ATM, MAPK1, MAPK3 |
| Colorectal cancer | 9 | 5.00E-05 | DCC, AKT1, CCND1, BRAF, TP53, PIK3CA, PIK3R1, MAPK1, MAPK3 |
| Measles | 9 | 1.20E-03 | AKT1, CCND1, IL2RA, EIF3H, TP53, PIK3CA, NFKB1, HSPA1A, PIK3R1 |
| Non-small cell lung cancer | 9 | 2.40E-04 | AKT1, EGFR, CCND1, BRAF, TP53, PIK3CA, PIK3R1, MAPK1, MAPK3 |
| Acute myeloid leukemia | 9 | 2.40E-04 | AKT1, CCND1, BRAF, FLT3, PIK3CA, NFKB1, PIK3R1, MAPK1, MAPK3 |
| Central carbon metabolism in cancer | 9 | 4.90E-04 | AKT1, EGFR, FLT3, TP53, PIK3CA, PTEN, PIK3R1, MAPK1, MAPK3 |
| ErbB signaling pathway | 9 | 2.50E-03 | AKT1, EGFR, BRAF, PAK2, MAP2K4, PIK3CA, PIK3R1, MAPK1, MAPK3 |
| HIF-1 signaling pathway | 9 | 4.00E-03 | AKT1, EGFR, SERPINE1, PIK3CA, NFKB1, GAPDH, PIK3R1, MAPK1, MAPK3 |
| TNF signaling pathway | 9 | 6.80E-03 | AKT1, NOD2, CASP7, MAP2K4, PIK3CA, NFKB1, PIK3R1, MAPK1, MAPK3 |
| Thyroid hormone signaling pathway | 9 | 9.60E-03 | AKT1, CCND1, TP53, ESR1, PIK3CA, MED14, PIK3R1, MAPK1, MAPK3 |
| Neurotrophin signaling pathway | 9 | 1.20E-02 | AKT1, BRAF, MAP3K1, TP53, PIK3CA, NFKB1, PIK3R1, MAPK1, MAPK3 |
| Bladder cancer | 8 | 4.30E-04 | EGFR, CCND1, BRAF, TP53, CDH1, THBS1, MAPK1, MAPK3 |
| Prolactin signaling pathway | 8 | 5.20E-03 | AKT1, CCND1, ESR1, PIK3CA, NFKB1, PIK3R1, MAPK1, MAPK3 |
| Apoptosis | 7 | 4.10E-04 | AKT1, CASP7, TP53, PIK3CA, NFKB1, PIK3R1, ATM |
| Small cell lung cancer | 7 | 2.20E-03 | AKT1, CCND1, TP53, PIK3CA, NFKB1, PTEN, PIK3R1 |
| mTOR signaling pathway | 7 | 1.30E-02 | AKT1, BRAF, PIK3CA, PTEN, PIK3R1, MAPK1, MAPK3 |
| Thyroid cancer | 6 | 1.00E-02 | CCND1, BRAF, TP53, CDH1, MAPK1, MAPK3 |
