# Supplementary figures and images for: Deep learning assisted multi-omics integration for survival and drug-response prediction in breast cancer
Source: BMC Genomics. 2021 Mar 24;22:214. doi: 10.1186/s12864-021-07524-2 (PMC7992339; doi:10.1186/s12864-021-07524-2)

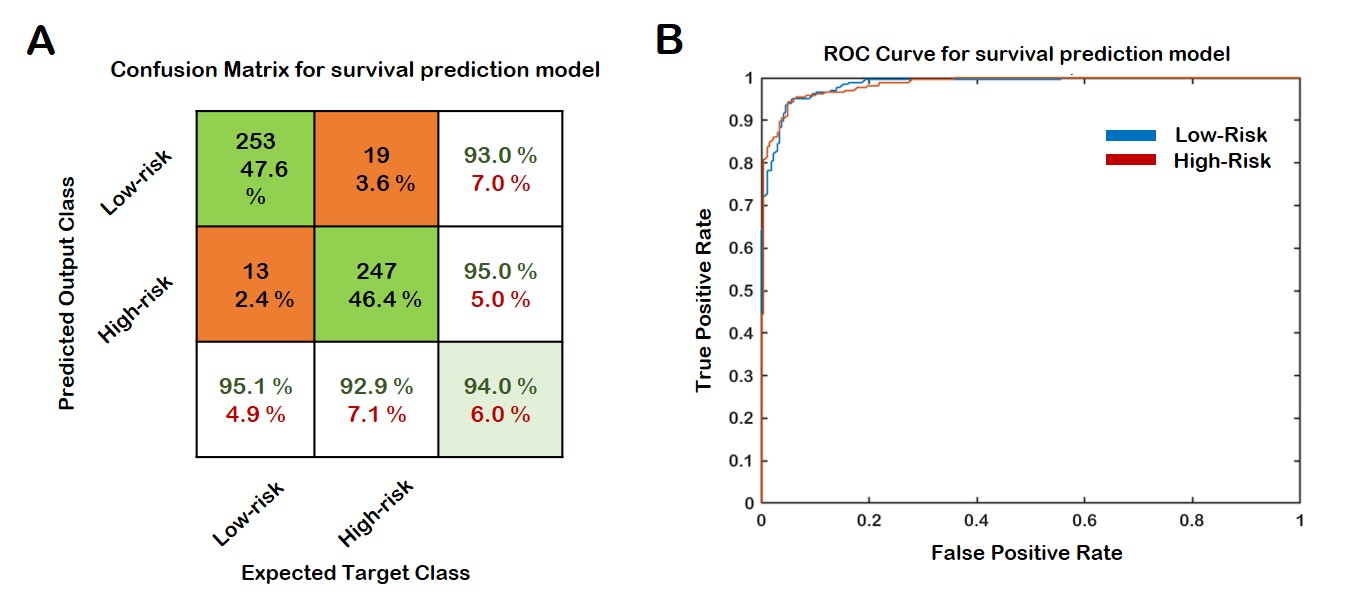

Supplement: Supplementary file 2 — Additional file 2: Figure S1. Performance of multi-omics integration based neural network survival prediction model of BRCA patients. (A) Confusion matrix and (B) ROC plot of neural network prediction model. [file 12864_2021_7524_MOESM2_ESM.jpg]

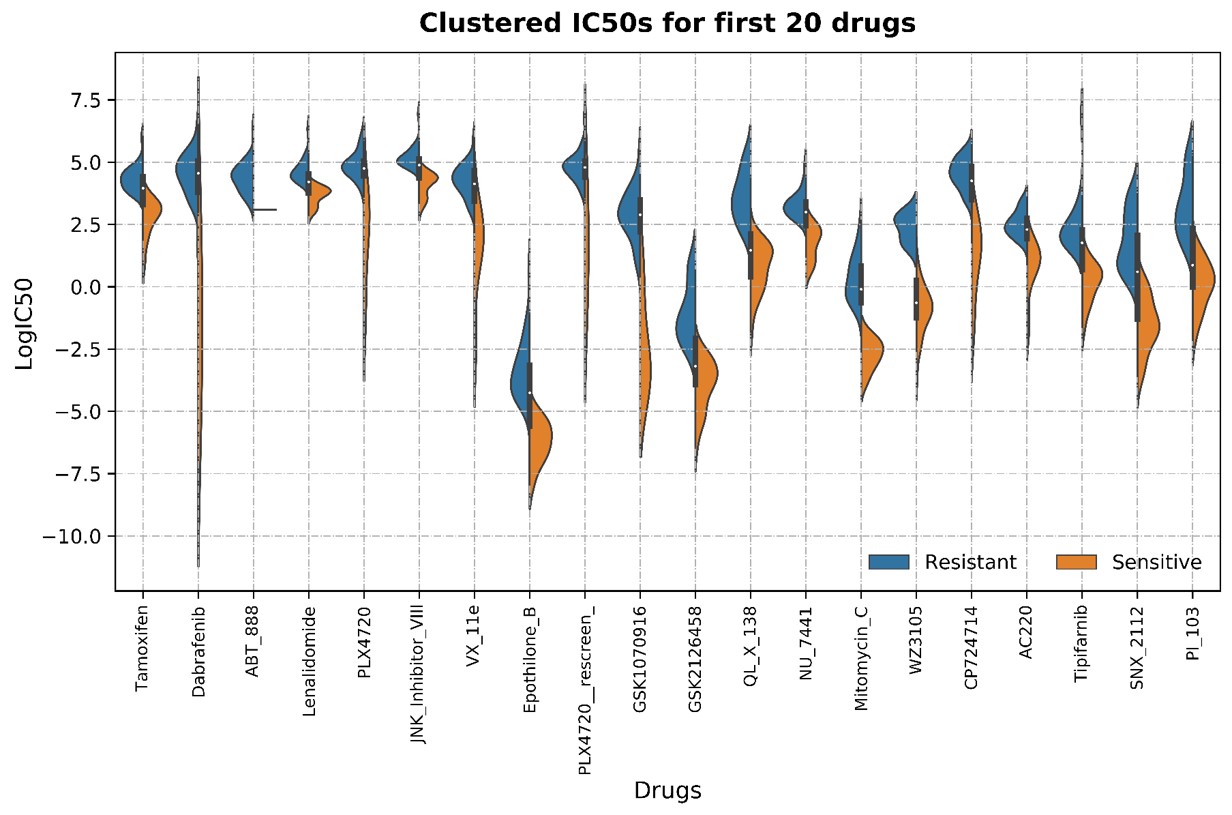

Supplement: Supplementary file 3 — Additional file 3: Figure S2. Violin plot showing the distribution of IC50 values of sensitive (blue) and resistant (orange) cell lines for the first 21 drugs. [file 12864_2021_7524_MOESM3_ESM.jpg]

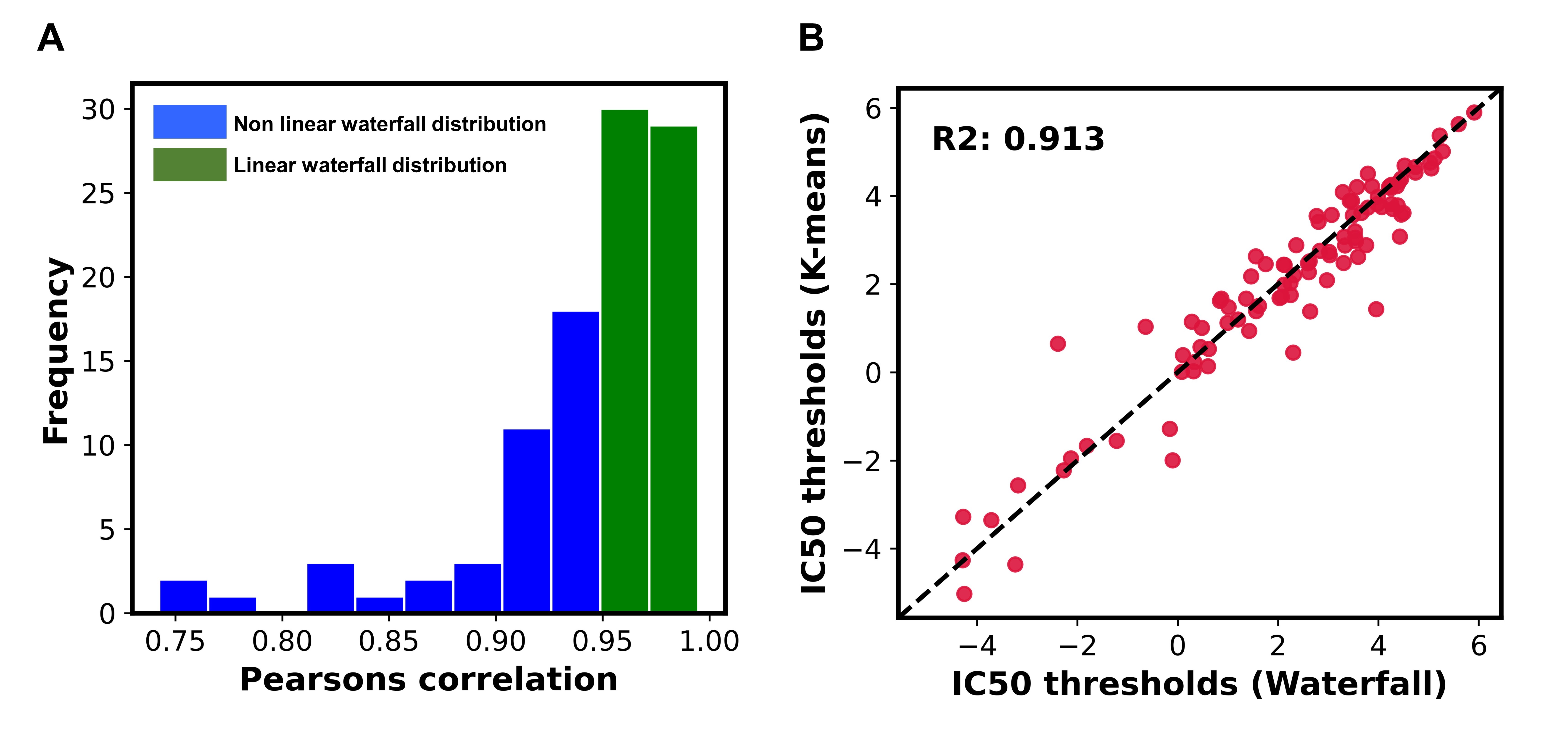

Supplement: Supplementary file 4 — Additional file 4: Figure S3. (A) Histogram depicting drugs with linear and non-linear correlation to a linear fit. (B) Correlation of IC50 thresholds calculated from the two methods (K-means and waterfall) shows that the two methods have consistent results. [file 12864_2021_7524_MOESM4_ESM.jpg]

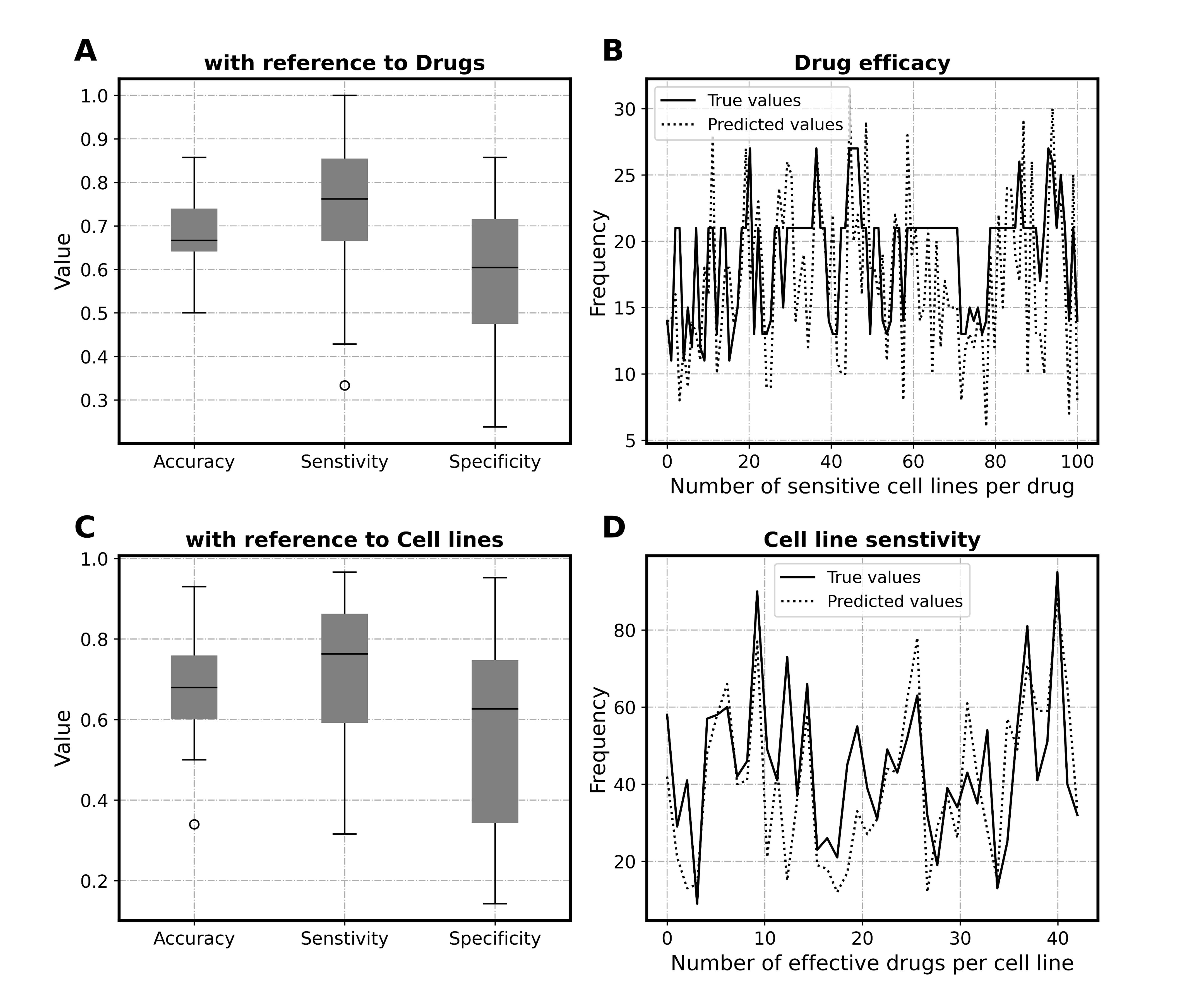

Supplement: Supplementary file 5 — Additional file 5: Figure S4. Performance of drug response model using thresholds from waterfall analysis. Box plot showing accuracy, sensitivity, and specificity of model for all drugs (A) and all cell lines (C). Scatter plot showing frequency of (B) sensitive cell lines per drug molecule and (D) effective drugs for each cell line. [file 12864_2021_7524_MOESM5_ESM.jpg]

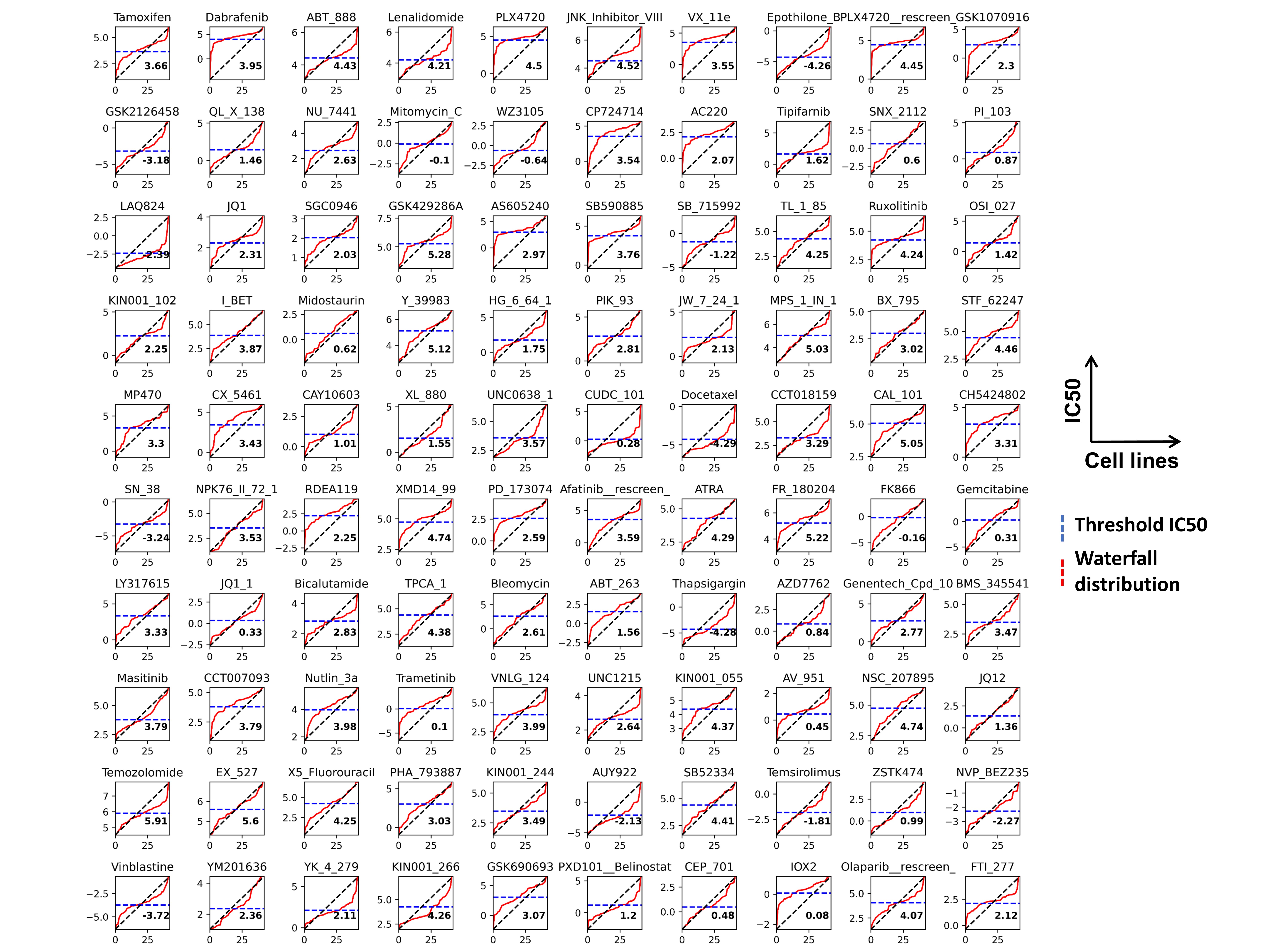

Supplement: Supplementary file 6 — Additional file 6: Figure S5. Waterfall distribution for 100 drugs under considerations. Blue lines depict IC50 thresholds as calculated by inflection point and median (threshold IC50 value is also depicted on each plot). [file 12864_2021_7524_MOESM6_ESM.jpg]
